# Mining Gut Microbiota From Bariatric Surgery for MAFLD

**DOI:** 10.3389/fendo.2021.612946

**Published:** 2021-04-09

**Authors:** Wei-Kai Wu, Yi-Hsun Chen, Po-Chu Lee, Po-Jen Yang, Chin-Chen Chang, Kao-Lang Liu, Cheng-Chih Hsu, Chi-Chang Huang, Hsiao-Li Chuang, Lee-Yan Sheen, Chun-Jen Liu, Ming-Shiang Wu

**Affiliations:** ^1^ Department of Medical Research, National Taiwan University Hospital, Taipei, Taiwan; ^2^ Department of Internal Medicine, National Taiwan University Hospital, Taipei, Taiwan; ^3^ Department of Internal Medicine, National Taiwan University College of Medicine, Taipei, Taiwan; ^4^ Department of Surgery, National Taiwan University Hospital, Taipei, Taiwan; ^5^ Department of Medical Imaging, National Taiwan University Hospital, Taipei, Taiwan; ^6^ Department of Chemistry, National Taiwan University, Taipei, Taiwan; ^7^ Graduate Institute of Sports Science, National Taiwan Sport University, Taoyuan, Taiwan; ^8^ National Laboratory Animal Center, National Applied Research Laboratories Research Institute, Taipei, Taiwan; ^9^ Institute of Food Science and Technology, National Taiwan University, Taipei, Taiwan

**Keywords:** MAFLD, bariatric surgery, gut microbiota, gut–liver axis, multi-omics, human microbiota associated mice, portal vein

## Abstract

The progression of metabolic dysfunction associated fatty liver disease (MAFLD) leads to steatohepatitis, liver fibrosis and hepatocellular carcinoma. Thus far, there have been no FDA-approved medications for MAFLD. Bariatric surgery (BS) has been found to improve insulin resistance, steatohepatitis and liver fibrosis but is not recommended for treating MAFLD due to its invasiveness. Recent studies suggest the improved glucose metabolism after BS is a result of, at least partly, alterations to the gut microbiota and its associated metabolites, including short chain fatty acids and bile acids. It makes sense the improved steatohepatitis and fibrosis after BS are also induced by the gut microbiota that involves in host metabolic modulation, for example, through altering bile acids composition. Given that the gut–liver axis is a path that may harbor unexplored mechanisms behind MAFLD, we review current literatures about disentangling the metabolic benefits of MAFLD after BS, with a focus on gut microbiota. Some useful research tools including the rodent BS model, the multiomics approach, and the human microbiota associated (HMA) mice are presented and discussed. We believe, by taking advantage of these modern translational tools, researchers will uncover microbiota related pathways to serve as potential therapeutic targets for treating MAFLD.

## Introduction

With a 25% global prevalence rate, metabolic dysfunction associated fatty liver disease (MAFLD), formerly named nonalcoholic fatty liver disease (NAFLD) is currently the most common chronic liver disease worldwide (1–3). In recent years, MAFLD has been getting attention since it has become the fastest growing etiology of liver cirrhosis and liver cancer worldwide (4, 5). Thus far, MAFLD has been the second-most common indication for liver cancer transplantation and the fastest growing indication in the United States (6), Furthermore, the number of deaths and end-stage liver disease caused by MAFLD is estimated to double from 2016 to 2030; global prevalence of MAFLD will continue to grow and data suggest the largest increase in MAFLD burden is expected to occur in Chinese population because of the urbanized lifestyle (5, 7).

MAFLD had been traditionally categorized into dichotomous simple steatosis and steatohepatitis based on the degree of histological severity of hepatic inflammation. Nevertheless, it is commonly accepted that MAFLD activity is a dynamic continuum and should be described by the grade of activity and the stage of fibrosis rather than a dichotomous stratification (2, 3). Thus far, the underlying pathogenesis in the progression of MAFLD is not fully understood. Although a “two-hit” theory for steatohepatitis has been proposed decades ago, the transition in the activity of MAFLD is a multifactorial continuum which involves with increased free fatty acids, translocated lipopolysaccharide (LPS), *de novo* lipogenesis, insulin resistance, oxidative stress, endoplasmic reticulum stress, mitochondria dysfunction, NLRP3 inflammasome activation, and inflammatory cytokines production such as IL-1β and IL-6 (8, 9). Chronic injuries to the liver parenchyma can lead to repeated necrosis and repairment and subsequently lead to liver fibrosis, cirrhosis, and hepatocellular carcinoma (HCC). However, the weight and importance of each mechanistic pathway is unknown and potential interactions between the multiple hits of MAFLD remain to be elucidated (9). Besides, the pathogenesis involves complex intercellular crosstalk between hepatocytes, Kupffer cells, hepatic stellate cells (HSCs) and other adaptive immune cells, further complicated the signal transduction of MAFLD (10, 11). Due to a lack of comprehensive knowledge, unfortunately, there are currently no FDA-approved drugs for the treatment of MAFLD on the market despite Vitamin E and pioglitazone as approved medications for selected patients in current guideline (12). Currently, the only well-validated measure for MAFLD treatment is lifestyle modification to achieve 7-10% weight reduction, however, only a minor portion of patients were able to sustain the weight loss over time (12).

Accumulating evidence have suggested that the gut microbiota can stand as an endocrine organ that is involved in dynamically regulating energy homeostasis and immune response in the human body (13). Actually, human organ systems could be affected when microbe-derived molecules interact with the host on the intestinal surface or react distally when transmitting across the intestinal barrier (14). The first stop in the human body receiving signals from the gut lumen is the liver, whereas the portal vein is the primary route for the blood returning from the gastrointestinal tract. Bioactive metabolites produced in the gut could travel from the intestinal lumen to the liver parenchyma through the portal vein as a fast track, which is known as the gut–liver axis. This pathway may allow the harboring of messages from the gut microbiota that are associated with the pathophysiological process in MAFLD (15, 16). In fact, links have been discovered between several microbial-produced molecules and the pathogenesis of MAFLD. These molecules include LPS, flagellin, peptidoglycan, and bacterial DNA which react with the toll-like receptor family (16, 17). Importantly, the short chain fatty acids (SCFAs) and bile acids are essential microbial-related metabolites that may regulate metabolic fate of MAFLD. Other small microbial molecules, including ethanol, trimethylamine (TMA), phenylacetate, and imidazole propionate, are also reportedly linking to the MAFLD (16, 18). Therefore, mining biological mass from the gut–liver axis could be a promising approach to discovering the mechanisms behind MAFLD progression and to generating novel therapeutic targets for treating MAFLD.

Bariatric surgery is a complex surgical procedure performed on morbidly obese patients to achieve weight reduction effectively. The most commonly practiced bariatric procedures are the sleeve gastrectomy (SG) and Roux-en-Y gastric bypass (RYGB). The SG involves the surgical resection of the fundal part of the stomach, while the RYGB is performed by creating a pouch from the stomach and connecting a newly created pouch to the small intestine. Both surgical operations involve stomach volume reduction, reduced acid production, and altered gut hormones, whereas RYGB involves the anatomical rearrangement of the gastrointestinal tract with an altered enterohepatic cycle that may affect bile acids reabsorption (19). Over the past two decades, bariatric surgery has been successfully used to treat or even cure adult-onset diabetes mellitus (DM) and has been proven the most effective therapy for DM (20–22). Although some important glucoregulatory roles of gut hormones have been firmly established, the physiological and molecular mechanisms underlying the benefits of bariatric surgery remain poorly understood (22). Recent data have also demonstrated that bariatric surgery can induce the dramatic improvement of MAFLD and reverse pathological features, including steatohepatitis and liver fibrosis (23, 24). Despite its significantly observed therapeutic effect for MAFLD, bariatric surgery is still not recommended for treating MAFLD as it is considered too invasive and cost-prohibitive (12). Therefore, disentangling the mechanisms behind how bariatric surgery treat MAFLD effectively may pave a new path in discovering potential therapeutic target for MAFLD. However, like the unsolved mechanisms behind bariatric surgery for DM, its beneficial mechanism in MAFLD remains largely unknown.

**Figure 1:**
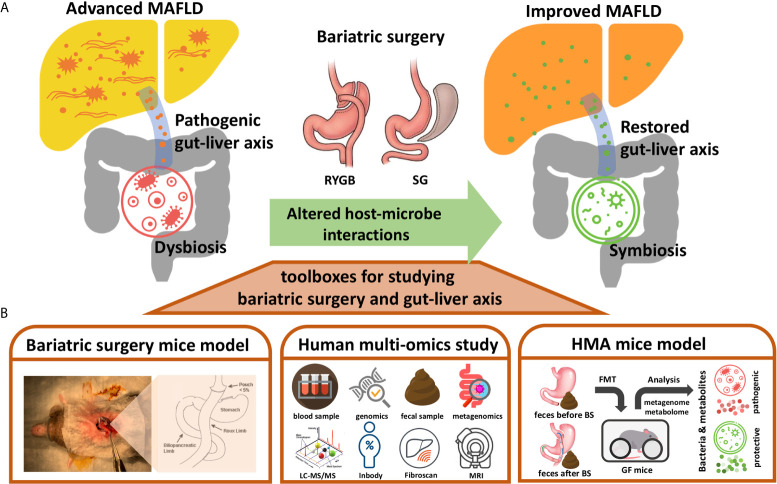
Toolboxes for investigating the benefits of bariatric surgery though gut-liver axis. **(A)** Diagram illustrating the metabolic benefits of bariatric surgery (BS) for MAFLD could be contributed by the altered host-microbe interactions. The pro-inflammatory gut-liver axis from dysbiosis might be restored by the bariatric surgery to improve MAFLD. **(B)** Approaches presented for studying the altered gut-liver axis by bariatric surgery and its potential beneficial effects on MAFLD, including bariatric surgery mice model, human multi-omics study, and human microbiota associated (HMA) mice model. RYGB, Roux-en-Y gastric bypass; SG, sleeve gastrectomy; LC-MS/MS, Liquid chromatography-tandem mass spectrometry; Inbody, a machine used for measuring human body composition; Fibroscan, a machine used for measuring stiffness of liver; MRI, magnetic resonance imaging; FMT, fecal microbiota transplantation.

Bariatric surgery usually involves the restriction of food intake in reducing energy absorption to achieve weight reduction. RYGB also alters the path of the digestive tract so that foods bypass the duodenum and upper jejunum to reduce the digestion and absorption of nutrients. The anatomical changes to the digestive tract resulting from bariatric surgery could have dramatic environmental impacts on the microorganisms living in the gut (19). The structure of the gut microbial community is usually rebuilt based on the environments changed in the gut lumen. For examples, reduced gastric acid production may permit more acid-intolerant bacteria to colonize the intestine (19). The RYGB shortens the distance for the bile salts to reach terminal ileum and colon, thus producing a bile acid abundant environment to the lower intestine and colon which affects both the composition and function of the gut microbiota, alter the primary/secondary bile acid ratio, and influence the conjugation process of bile acid in the gut lumen (25). In addition, since the RYGB bypasses the gastroduodenal portion, more oxygen is swallowed into the small intestine, thereby extending the intestinal tract as a microaerobic rather than an anaerobic state (26). The altered oxygen gradient along the intestinal tract by RYGB allows more aerotolerant bacteria to colonize in the colon, such as *Escherichia coli*, *Streptococcus* spp. and *Veillonella* spp (27). In contrast, some relative abundance of anaerobes, such as Clostridium spp., was observed to be higher after SG which is probably caused by less swallowed oxygen to the reduced gastric volume. However, a potential anti-obesity and anti-diabetic anaerobic bacterium, *Akkermansia muciniphila*, was enriched after both surgeries (27, 28). Interestingly, some gut microbe-associated glucoregulatory hormones such as GLP-1 and FGF19, were proposed as a mechanistic route of bariatric surgery for improving DM (19, 22). Therefore, it would be no surprise if at least some mechanisms underlying the benefits of bariatric surgery for MAFLD were the results from the alterations of gut microbiota. In this mini-review, we present some potentially applicable approaches such as bariatric surgery mice model, human multiomics study, and human microbiota associated (HMA) mice study for investigating the benefits of bariatric surgery through the gut microbiome–liver axis (**Figure 1**), and discuss current gaps and challenges that researchers need to face.

## Mining With Rodent Model of Bariatric Surgery

The animal model of bariatric surgery has been developed since early 1990s for learning bariatric surgical techniques and understanding the postsurgical physiology of bariatric surgery (29). Over the past decade, the experimental rodent model for both gastric bypass surgery and SG have been matured and well validated, thus providing a delicate research tool for studying the molecular mechanisms behind the metabolic fates from bariatric surgery (30). Importantly, a rodent model for bariatric surgery is useful for researchers to investigate whether the metabolic benefits such as improved glucose tolerance and steatohepatitis could be caused by other drastic physiological changes (including alterations to gut microbiota) induced by bariatric surgery, instead of being caused by the expected weight reduction. For example, Liou et al. found the *Escherichia coli* and *Akkermansia muciniphila* were increased when mice received RYGB surgery and found the transfer of feces from RYGB-treated mice to germ-free mice significantly induced weight reduction, decreased fat mass, and improved glucose tolerance which were linked to decreased acetate and increased propionate in the feces (31). The gastric bypass surgery may also benefit by improving the gut barrier. Yang et al. performed duodenojejunal bypass (DJB) surgery in rodent animal and found a strengthened gut barrier and increased serum GLP-2 in rats that received DJB when comparing to the sham control group (32). These studies support the murine model of bariatric surgery to serve as a promising tool in disentangling the beneficial mechanisms behind the surgery-altered gut microbiota. For the SG procedure, as it does not alter the digestive path, the alterations to the gut microbiota post-SG may be less drastic than that in RYGB. However, the compositional and functional changes to the gut microbiota caused by SG remain important in modulating the metabolic fate in the post-SG host. For example, Ryan et al. conducted a vertical sleeve gastrectomy (VSG) mice study and demonstrated that a part of the weight-reducing effects of SG was not the result of the intake restriction imposed by a smaller stomach but rather the effect of the altered level of bile acids and the changes in the gut microbiota (33). Further, they showed that the weight-reducing effects of the VSG in mice were substantially reduced when the FXR, an essential bile acid receptor gene, was knocked out. Collectively, current evidence suggests that changes to the gut microbiota after bariatric surgery are not just a consequence of an altered intestinal environment but also a contributor to the metabolic benefits. Therefore, it provides an opportunity for researchers to investigate whether MAFLD could be improved through the altered gut microbiota caused by bariatric surgery.

## Mining in Human Multi-Omics Study

With the rapid advancement of new technology such as next generation sequencing and mass spectrometry, the translational research has entered into a new era as growing volumes of omics data have been generated. The integration of multiomics data from both host and its harboring microbes which include 16S rRNA taxonomics, shotgun metagenomics, metatranscriptomics, metaproteomics, metabolomics and the clinical phenotypic health datasets, has enabled efficient investigations on disease-related host-microbe relationships (34, 35). Recently, the multi-omics approach is becoming popular for exploring the microbial and molecular features of disease phenotypes, including MAFLD (36). For example, Hoyles et al. analyzed samples from 105 morbidly obese women by integrating features of the liver transcriptome, plasma and urine metabolome, and gut metagenome. The authors established molecular networks linking the gut microbiome to hepatic steatosis and concluded that the gut microbiome has substantial effects on the human steatosis phenotype through producing specific metabolites such as phenylacetic acid and 3-(4-hydroxyphenyl) lactate (37). Tremaroli et al. integrated shotgun metagenome sequencing and targeted metabolomics to investigate the gut microbiome changes caused by bariatric surgery and found that both RYGB and vertical banded gastroplasty produce increased postprandial levels of FGF19 (a downstream effector of FXR receptor) and durable alterations to the gut microbiome, which are independent of the BMI (38). Interestingly, they colonized feces from human donors to the germ-free mice and showed a favorable fat mass regulation contributed by the surgically altered microbiota. Aron-Wisnewsky et al. conducted a larger multi-omics study to investigate the structural and functional effects of bariatric surgery on the gut microbial community (39). The authors analyzed the time-series gut microbiome and serum metabolomics among 61 morbidly obese patients who received bariatric surgery and found that 75% of them had low microbial gene richness, which partly restored after the bariatric surgery. By integrating serum metabolomics and fecal metagenomics data, they found that nine potential microbe-derived metabolites in the serum, including glutarate, 3-methoxyphenylacetic acid, and L-histidine, are strongly correlated with low microbial gene richness, which might provide functional clues to disentangle the altered microbial features induced by bariatric surgery. More recently, Farin et al. conducted a metagenome study comparing the impact of RYGB and SG on gut microbiota. Both RYGB and SG increased alpha diversity and gene richness of gut microbiota while the microbiome composition is differently altered (27). The RYGB promotes colonization of aerotolerant oral flora such as *Streptococcus* and *Veillonella* spp. more than SG. At the functional level, the module of propionate production was higher after RYGB which is consistent with previous animal study conducted by Liou et al. (31). Other recent studies comparing gut microbiome after bariatric surgery by using 16S rRNA amplicon sequencing showed a decreased *Firmicutes*/*Bacteroidetes* ratio (40, 41), indicating a favorable phylum composition for obesity which is consistent with a recent meta-analysis showing increase in phyla Bacteroidetes, Fusobacteria, Proteobacteria and Verrucomicrobia, and a decrease in Firmicutes (28).

Analysis of metabolomics from samples of peripheral blood may not be a perfect way to explore the host-microbe communications among gut–liver axis because most microbe-derived molecules from the intestine converge into liver through the portal vein, leveraging an effect called first-pass metabolism (42). The molecules entering the liver could be readily inactivated, modified, or consumed by hepatic cells, so the composition and concentration of microbe-derived metabolites observed in the portal vein may be largely different when entering into systemic circulation. Thus, the portal vein is considered an optimal place for mining unbiased microbial signals from the gut–liver axis. Koh et al. took advantage of this concept to explore signals transmitted from the gut microbiota to the host by sampling portal veins from five obese DM patients and 10 BMI-matched DM-negative obese subjects who received bariatric surgery (43). The authors found four amino acid-derived metabolites (dopamine sulfate, glutamate, imidazole propionate, and N-acetylputrescine) that were significantly elevated in the portal vein of the DM patients and finally proved the microbe-produced imidazole propionate to be a novel etiology for impaired glucose tolerance. Thus, we believe it plausible to discover new microbial-derived therapeutic targets for MAFLD by using similar approach.

### Mining in Human Microbiota-Associated Mice Research

The establishment of human-sourced gnotobiotic mouse model through the fecal microbiota transplantation (FMT) of human donors into germ-free mic has provided an innovative and plausible tool in mimicking the human microbial system (44, 45). Although the mouse model allows the perturbation in the gut microbiota by bariatric surgery to be studied with a controlled experimental setup and has demonstrated the causal effects of gut microbiota modulated by bariatric surgery for improving metabolic disorders, challenges remain. Before the research findings can be translated to clinical application, the substantial differences in the compositions of the gut microbial communities between humans and rodents caused by host–microbe selectivity must be addressed (46). Thus, a model for human microbiota-associated (HMA) mice, also named humanized gnotobiotic mice, was created to fill the gap between rodent and human gut microbiota studies. Basically, the HMA was created by transplanting feces from human donors to germ-free recipient mice or antibiotic-treated mice to investigate whether the disease or therapeutic phenotypes observed in the human study could be transferred to controlled experimental mice by colonizing the phenotype-associated microbiota (47). For example, Hoyles et al. demonstrated the role of human gut microbiota in *de novo* lipogenesis by transplanting feces from patients with different grades of liver steatosis to antibiotic-treated mice. In this study, they further discovered that phenylacetic acid, a metabolite derived from the microbial metabolism of phenylalanine, mechanistically triggers liver steatosis (37). Besides, the human-sourced gut microbiota transplanted to mice can be manipulated by different antibiotics to screen for the optimal microbial composition that contributes either positively or negatively to the phenotype of interest. Tanoue et al. used antibiotics of different spectrums on HMA mice and successfully isolated a consortium of 11 bacterial strains that robustly induced interferon-γ-producing CD8 T cells in the intestine (48). Therefore, we consider the HMA mice model a potential tool in mining therapeutic targets for MAFLD from the gut–liver axis altered by bariatric surgery.

## Common Changes of Gut Microbiota After Bariatric Surgery

Datasets related to the microbiome change after bariatric surgery have been accumulating in recent years. Although the changes of gut microbial composition may differ based on different operation types of bariatric surgery, increased microbial diversity and gene richness were generally observed in most studies (49). Several potentially beneficial anaerobic commensal bacteria such as *Akkermansia muciniphila*, *Rosburia intestinalis*, and *Faecalibacterium prausnitzii*, has been reported to be enriched after bariatric surgery (49). The increased relative abundances of these bacteria may contribute to the weight reduction and the improved metabolic fates after bariatric surgery although the causality and mechanisms require to be proved. Notably, some opportunistic pathogen belonging to *Proteobacteria* including *Escherichia coli* and *Klebsiella* were increased after bariatric surgery and the effects also need to be followed. Nevertheless, these results might be limited and require to be interpretated carefully because the sample size of current studies are relatively small, and in most occasions, used 16S rRNA sequencing for microbiome profiling which has relatively low resolution for microbial taxonomy. Importantly, whether the change of gut microbiota caused by bariatric surgery is a key to the improvement of metabolic syndrome remains to be elucidated. Interestingly, a recent pilot study was conducted by transplanting fecal microbiota from donors who received RYGB to subjects with metabolic syndrome (50). The authors found a relatively increased insulin sensitivity by fecal microbiota transplantation (FMT) from RYGB donors as compared with donors having metabolic syndrome. However, the study has small sample size and several confounding factors, thus the metabolic effects of FMT from donors receiving bariatric surgery remain inconclusive and need to be elucidated with a larger and less-biased study.

## Bariatric Surgery, Bile Acids and MAFLD

Given that the mechanisms of bile acids in regulating glucose and lipid metabolism have been established, the alteration in bile acids composition after bariatric surgery is considered an explanation to its metabolic effects (25, 51). Briefly, the primary bile acids (PBA) are synthesized and conjugated in the liver, excreted to the intestine, deconjugated and/or transformed to secondary bile acids (SBA) by the gut microbiota, and recycled through reabsorption in the terminal ileum (51). The RYGB has foods bypass the gastroduodenal portion, reducing consumption of bile acids, and creates a shortcut for bile acids to reach the lower intestine (19). This anatomical change may have more conjugated bile acids to be actively reabsorbed in terminal ileum while parallelly allow more PBA to enter into colon to be transformed to SBA by the gut microbiota. Studies have showed that in MAFLD patients, the PBA/SBA ratio in plasma were significantly higher when comparing with healthy subjects and correlates to the severity of MAFLD (51, 52). Interestingly, current data also showed the bariatric surgery consistently decrease the PBA/SBA ratio which might be related to the metabolic benefits of MAFLD by bariatric surgery (51). However, it should be noted the bile acids have numerous chemical structures which exhibits quite different affinity to FXR and TGR5 and represent divergent downstream bioactivities, making interpretations of the altered bile acids composition more complicated (25). Nevertheless, the simple plasmatic PBA/SBA ratio might still be a potential clinical indicator for predicting the improvement of MAFLD by bariatric surgery but still need to be validated in a larger cohort study. Interestingly, a more recent study showed a gut microbiota-dependent pathway by which SG increases liver cholic acid-7-sulfate (CA7S) production and subsequent TGR5 signaling and GLP-1 production (53). This authors further demonstrated liver CA7S production was induced by increased lithocholic acid (LCA), a secondary bile acid produced by *Clostridia*, transported selectively from gut lumen into portal vein, thus illustrating CA7S as a microbiota-dependent metabolite altered by bariatric surgery and is responsible, at least partly, for the metabolic improvement (including MAFLD) of bariatric surgery (54). Notably, these findings were demonstrated by mouse model and requires to be validated in future human study.

## Discussion

In this review paper, we presented some experimental approaches that are potentially useful for exploring the link between gut microbiota and metabolic fates through the gut–liver axis. We believe that these approaches could be useful for investigators to study the mysterious benefits of bariatric surgery for MAFLD and to decipher potential protective mechanisms that are relevant to the altered gut microbiota. By investigating the gut–liver axis, researchers may have an opportunity to discover new therapeutic targets from the metabolic benefits of bariatric surgery, which is so far the most effective treatment for MAFLD. However, despite being promising, some challenges and limitations remain.

First, the improvement of MAFLD usually correlates well with weight reduction, making it vague whether the improvement of MAFLD after bariatric surgery is caused by the expected weight loss or by the altered gut microbiota, especially in clinical research. Studies conducted with time-series observations for changes in MAFLD-relevant biomarkers and the alterations in the gut microbiota might be helpful for clarifying this ambiguity. Ooi et al. conducted an observational study with monthly follow-ups with 84 morbidly obese patients who received bariatric surgery to collect anthropometric and serological data for 12 months (55). In this study, serum alanine aminotransferase and γ-glutamyl transferase dropped faster and greater than improvements in body weight, serum triglyceride, and glucose within three months after the bariatric surgery. The observation showing hepatic inflammation to be attenuated earlier than the occurrence of weight reduction suggests that the improvement of liver injuries may be mechanistically independent from weight loss, while both are the results of bariatric surgery. Although improved serum liver enzymes may not indicate histologically improved MAFLD, the serological finding is consistent with two other clinical studies that have demonstrated a significant histological improvement in MAFLD within 3–6 months after bariatric surgery (56, 57). The early therapeutic response of MAFLD to bariatric surgery may correlate with the rapid adaptation of the gut microbiota to a new gut environment created by the operation. Besides, rodent studies have also shown the role of gut microbiota modulated by bariatric surgery in improving metabolic fates (31, 33). Collectively, both clinical and basic evidence provide reasons to believe that the improvement of MAFLD after bariatric surgery could be induced by an unsolved mechanism that works through the altered gut–liver axis.

Although the gut microbial community is undoubtedly affected by bariatric surgery, the prophylaxis antibiotic used during the operation and the altered dietary habits after surgery also significantly impact the gut microbiota, which increases the inter-individual variations for an altered gut microbiome (19). Therefore, the study sample size may need to be augmented to adjust for the variations produced by these unavoidable confounders. However, the follow-up for the MAFLD phenotype histologically is a challenging task. Hence, applying non-invasive tools such as magnetic resonance imaging proton density fat fraction (MRI-PDFF), Fibroscan, and magnetic resonance elastography (MRE), would be necessary for implementing a large-scale clinical study to compare MAFLD severity before and after bariatric surgery. For example, Caussay et al. compared the gut microbial signatures among patients with MAFLD at different fibrosis stages by using MRI-PDFF and MRE to quantify steatosis and fibrosis of the liver. They found 27 bacterial features to build a robust random forest classifier model for MAFLD-related cirrhosis with a prediction rate of 92% AUROC, which was confirmed by an independent validation cohort (AUROC of 0.87) (58).

Despite being challenging, investigation on the metabolic benefits brought about by bariatric surgery for MAFLD remains an exciting filed since the advancement in microbiome research has provided opportunities for deciphering the mechanisms behind previously unsolved diseases. As the liver is the sentinel organ in receiving microbial signals from the portal vein, we believe mining the altered gut-liver axis from bariatric surgery holds a great potential to bring new mechanistic insights for MAFLD.

## Author Contributions

W-KW conceived and drafted the paper. M-SW supervised and revised the study. Y-HC, P-CL, P-JY, C-CC, K-LL, C-CHs, C-CHu, H-LC, L-YS, C-JL provided professional insights to the paper. All authors contributed to the article and approved the submitted version.

## Funding

This work was supported by the Ministry of Science and Technology (Taiwan) (108-2314-B-002-032, 106-2314-B-002 -039-MY3 and 108-2321-B-002 -035, 106-2319-B-492-001, 109-2327-B-002 -005, 109-2314-B-002 -103 -MY3, 109-2314-B-002 -064 -MY3).

## Conflict of Interest

The authors declare that the research was conducted in the absence of any commercial or financial relationships that could be construed as a potential conflict of interest.
